# Hope and despair: community health assistants’ experiences of working in a rural district in Zambia

**DOI:** 10.1186/1478-4491-12-30

**Published:** 2014-05-25

**Authors:** Joseph Mumba Zulu, John Kinsman, Charles Michelo, Anna-Karin Hurtig

**Affiliations:** 1 Department of Public Health, School of Medicine, University of Zambia, P.O. Box 50110, Lusaka, Zambia; 2 Umeå International School of Public Health (UISPH), Umeå University, Umeå SE 90185, Sweden

**Keywords:** Community-based health workers, Work experience, Work motivation, Zambia

## Abstract

**Background:**

In order to address the challenges facing the community-based health workforce in Zambia, the Ministry of Health implemented the national community health assistant strategy in 2010. The strategy aims to address the challenges by creating a new group of workers called community health assistants (CHAs) and integrating them into the health system. The first group started working in August 2012. The objective of this paper is to document their motivation to become a CHA, their experiences of working in a rural district, and how these experiences affected their motivation to work.

**Methods:**

A phenomenological approach was used to examine CHAs’ experiences. Data collected through in-depth interviews with 12 CHAs in Kapiri Mposhi district and observations were analysed using a thematic analysis approach.

**Results:**

Personal characteristics such as previous experience and knowledge, passion to serve the community and a desire to improve skills motivated people to become CHAs. Health systems characteristics such as an inclusive work culture in some health posts motivated CHAs to work. Conversely, a non-inclusive work culture created a social structure which constrained CHAs’ ability to learn, to be innovative and to effectively conduct their duties. Further, limited supervision, misconceptions about CHA roles, poor prioritisation of CHA tasks by some supervisors, as well as non- and irregular payment of incentives also adversely affected CHAs’ ability to work effectively. In addition, negative feedback from some colleagues at the health posts affected CHA’s self-confidence and professional outlook. In the community, respect and support provided to CHAs by community members instilled a sense of recognition, appreciation and belonging in CHAs which inspired them to work. On the other hand, limited drug supplies and support from other community-based health workers due to their exclusion from the government payroll inhibited CHAs’ ability to deliver services.

**Conclusions:**

Programmes aimed at integrating community-based health workers into health systems should adequately consider multiple incentives, effective management, supervision and support from the district. These should be tailored towards enhancing the individual, health system and community characteristics that positively impact work motivation at the local level if such programmes are to effectively contribute towards improved primary healthcare.

## Background

The World Health Organization (WHO) has expressed concern that several low and middle income countries, including Zambia, face critical shortages of human resources for health (HRH) [1]. Several factors have contributed to this problem, with countries’ limited capacity to train staff being one of them. International migration has also been cited as a contributing factor for this crisis [2], with some workers migrating to other countries in search of professional development and a better quality of life [3]. The consequent shortage of staff has increased workloads and contributed towards de-motivation, while also greatly affecting the delivery of services in the affected countries [4], as well as health system development [5].

In an attempt to resolve these gaps, countries have come up with different strategies, one of these being the development of national, large-scale community-based health worker programmes [6]. These programmes engage the services of community members to deliver primary healthcare services at community level. The workers are recruited, trained, managed and paid by the government [7]. According to a comprehensive review conducted by the Global Health Workforce Alliance (GHWA) in 2010, the main national community health worker programmes include the Brazil’s Agentes de Sa´ude, Ethiopia’s Health Extension Worker (HEW), India’s Accredited Social Health Activist System (ASHAs) and the Lady Health Worker Program (LHW) in Pakistan [6].

Maternal and child health services are among the key services provided by the community-based health worker programmes [8,9]. The ASHAs in India specialise primarily in promoting institutional deliveries and universal immunization of children [7]. The LHW programme in Pakistan delivers vaccination to children and promotes the use of antenatal services and modern family planning methods, among other activities [6]. Similarly, the Ethiopian HEW programme provides antenatal care services and immunization programmes for children [10,9]. In addition to addressing maternal and child health matters, Brazil’s Agentes de Sa´ude also provides cancer screening services and facilitates the collection of vital statistics [8,11].

Several factors have shaped the performance of community-based health workers. Type and quality of supervision, level of linkages with health system structures, availability of drugs, clarity of the responsibilities, funding patterns, and quality of programme management are some of the factors that shaped their performance [7]. The 2006 World Health Report has identified the key factors that influence the performance of health workers within three broad categories, namely: characteristics of the health workers; the health system; and the population served by the health workers [4]. Performance is the result of a transactional process between health workers and their work and the community environment. It is the process through which workers engage with the conditions in the health system and community to provide healthcare according to their abilities, professional values and personal goals [12,13].

Motivation is a key factor affecting the performance of community-based health workers. The quality and type of supervision, and familiarity with the local work environment were shown to be important in shaping work motivation among the Agentes de Sa’ude in Brazil [7,11] as well as in the Lady Health Worker program in Pakistan [6]. Studies on the India's ASHAs showed that their work motivation was reduced by unclear understanding of their responsibilities, a lack of knowledge to perform the job as well as inconsistent financing [7]. Like the other community-based health workers, the Ethiopian HEWs also experienced limitations such as inadequate management capacity by supervisors and training, pay inconsistency and employment insecurity as well as limited infrastructure which affected their ability to work in the initial stages of the programme [10]. Another study on the Ethiopian HEWs identified the following as key factors for improving worker motivation: providing sufficient orientation to communities regarding their role; ongoing instruction and mentoring processes; strengthening teaching materials; giving special consideration for personal advancement; and involving and training leaders of community anchors to support and motivate community-based health workers [14].

Community-based health worker motivation has been categorised at four levels: individual; family; community; and organisational. At the individual level, the motivation relates to the possibility of applying knowledge gained to solve their families’, communities’ and their own problems. Families and communities facilitate work motivation in the form of moral, financial and material support. The organisational level provides motivation by providing stipends, potential employment, materials, training and supervision [15]. With regards to working in rural areas, several studies have identified professional ethos, and a desire to help the poor as some of the motivating factors [16-18]. Meanwhile, Gross *et al*. [19] classify determinants of performance among health workers in what they term as ‘workhood assets’ which includes social, physical and financial capital.

### The community health assistant strategy in Zambia

Zambia has about half the health workforce that it needs. As of 2010, vacancies among nursing cadres stood at 55%, clinical officers 63% and doctors 64% [20,21]. As a result, about 23,500 community-based health workers have been helping in providing primary healthcare (PHC) across the country. Community-based health workers are defined as ‘members of communities who work either for pay or as volunteers in association with the local health care system and usually share ethnicity, language, socio-economic status and life experiences with the community members they serve. They have many titles, including home based care givers (often work with faith-based programmes), health promoters, community health advisors, lay health advocates, community health representatives, and peer health educators’ [21]]. Community-based health worker activities include managing malaria fevers, ‘promoting proper food production, basic sanitation, and detecting risk groups for the prevention of common illness. In HIV prevention, treatment and care programs, they organize support groups and perform home visits for patients who miss their appointments, clearly greatly contributing to the supply of health service and in particular to the rapid expansion of antiretroviral drug delivery’ [22]. Community-based health workers either work for public health facilities, faith-based organisations or NGOs [21].

Community-based health workers have faced a number of challenges in discharging their duties, and their effectiveness has not always been optimal. The challenges include ‘low motivation, inadequate supervision, insufficient compensation or incentives, and low recognition by qualified health care providers’ [22]. In an attempt to address the country’s huge HRH gap, the Ministry of Health (MoH) developed a national community-based worker programme called the National Community Health Assistant (CHA) strategy in 2010. The MoH has established a Strategic Team to coordinate monitoring of the process of implementing the CHA strategy. The Team is also supposed to document the successes and challenges of the pilot phase and inform the scale-up process [21].

Compared to other community-based health workers, CHAs are recruited by the MoH, with the support of the community leaders and the Neighbourhood Health Committees (NHCs). The NHCs are community structures that help in managing and organising health promotion programmes at community level. Furthermore, unlike other community-based health workers, CHAs are registered by health professional bodies, are supervised by nurses, receive a standardised 1-year training, and will be placed on government payroll. CHAs are entitled to a monthly allowance of about 1,500 kwacha (about 220 dollars), which is almost one-third of the monthly salary of a comparable position, the nurse. CHAs also have a standardised work schedule, based on which they are expected to spend 80% of their work time in the community and 20% in the health posts [20]. The main sponsor of the CHA programme is the Department for International Development while the Clinton Health Access Initiative (CHAI) provides technical support to the MoH in implementing the programme [21,22].

Piloting of the CHA programme started in 2011. The first group of 307 CHAs were deployed after training in health posts in August 2012. CHAs are now operating in seven out of the nine provinces in the country (48 districts and 161 health posts) [23]. On average, two CHAs were deployed at each health post. A health post usually has one trained staff (nurse) [21], but with support staff (for example, cashiers, cleaners and guards) who also help with basic tasks, usually under the supervision of nurses. These support staff help with immunisations for children aged under 5 years as well as screening of patients. They learn mainly through the experience which they gain by working with professional health workers in conducting these tasks [20].

The activities of CHAs at the health posts include screening patients (taking vital signs), treating minor illnesses such as malaria, diarrhoea, respiratory tract infections and burns/sores, assisting with delivering children and counselling patients, which are the common health concerns in the districts were CHAs have been deployed [21]. In the community, CHAs conduct awareness campaigns on the use of mosquito nets as well as on sanitation, they help reduce occurrence of diarrhoea by encouraging community members to apply chlorine, and they test for and treat minor illnesses. CHAs also develop registers of community members. The registers show the total number of people and common diseases in the community. The registers guide the MoH in planning community health services [21].

Although, some countries have implemented programmes aimed at formalising community-based health workers, there is limited knowledge from low and middle income settings on their daily work experience and how their experiences shape their motivation to work. Recent studies on formalised community-based health workforces have focused on recruitment processes [23], their contribution towards disease-specific outcomes [24-26], cost control and logistical constraints [27], and programme management [7]. This paper intends to fill this knowledge gap by specifically exploring CHA experiences and how these affect their motivation to do a good job. This paper is a sub-study of a project on Integrating Community Health Assistants into the Health System in Zambia. Other components of the project explore community members as well as health centre staff’s perceptions of the CHAs.

### Methodology

#### The study design

The study takes a phenomenological approach, as it is ‘the participants’ perceptions, feelings, and lived experiences that are paramount and that are the objective of study’ [28]. Phenomenological research focuses on people’s daily life experiences as experienced. It is a meaning-oriented ‘approach and includes discovering, analysing, clarifying and seeking patterns of a certain phenomenon, based on a description of how the life world of humans is experienced, acted out and described. It presupposes striving to stay open to the studied phenomenon and its meanings’ [29]. A phenomenological approach is relevant for this study because its major focus was to describe and understand CHAs daily work experience. Further, we aimed to discover, analyse, clarify and seek patterns of a phenomenon: CHAs’ work experiences and how these affect their motivation to work.

#### Description of study site

Kapiri Mposhi district is in the Central Province of Zambia. It is a rural district located 185 kilometres north of the capital Lusaka. The district has one hospital, four health centres and 22 health posts. The district is demarcated into four main zones, each zone having one health centre. In 2010 it had a population of about 240,841, with an average annual population growth rate of about 2.1% [30].

Kapiri Mposhi district was purposively chosen for this study because it is one of the rural districts where the CHA strategy has been piloted and with easy access from Lusaka. The programme is being implemented here in six health posts, the lowest level of health service. Most of the health posts are far the from the main district hospital. Some of them are almost inaccessible during the rainy season (December to February), due to flooding. The health posts service no less than 3,000 people.

#### Sample size

Twelve CHAs who were deployed in the district in August 2012 were included in the study. This is the total number of CHAs deployed and working in the district at the time of the study. The CHAs were identified from the district’s human resources for health records.

### Data collection techniques

#### In-depth interviews

In conformity with the phenomenological approach, the main form of data collection was in-depth interviews, which were conducted from July 2013 to September 2013. In-depth interviews allow the researcher to gain an insight into the world of the participant, in this case the CHAs, in an open or unprejudiced way. This data collection technique enabled the CHAs to freely tell their stories about their daily realities, both at the health post and in the community.

Twelve in-depth interviews were conducted with all the CHAs in the district at the health posts separately by either the first, second or fourth authors, all of whom who have training and experience in qualitative research. The diverse backgrounds and qualifications of the researchers (anthropology and public health) helped in improving credibility of data. Credibility was also enhanced through the researchers’ complementary insights into the data collection process as well as the collected data. All the interviews were conducted in English as respondents were conversant with the language. An average interview lasted for about 1.5 hours. Interviews were recorded, but notes were also taken by researchers during the interviews, and these were extensively reviewed and discussed.

An interview guide developed by the authors was used during the data collection. Open-ended questions and inductive probing were adopted during the data collection process. This allowed us to clarify expressions or meanings of the CHAs’ daily experiences at the health posts and communities, and further permitted the CHAs to freely tell their stories. The questions focused on the following key issues: processes of becoming a CHA; CHA skills; activities; supervision; and general successes and challenges experienced by CHAs.

### Observations

We conducted observations at all health posts. These involved sitting at the health post and discussing with CHAs and other staff while paying attention to involvement of CHAs in the activities at the health post such as screening process of patients and participation in weekly and technical support meetings. Each health post was observed on three different occasions and each observation lasted for about half a day. Observations were also conducted on the availability of supplies at the health posts. Issues reported by CHAs regarding level of involvement in tasks and meetings at the health posts were found to be consistent with what was observed. However, it was not possible to cross-check using observations the issues raised by CHAs regarding inter-personal interactions, as these required a longer observational time.

### Review of documents

We reviewed documents and reports related to the implementation of CHAs in Zambia. The documents were identified and actively searched for by the authors at national and district offices as well as at the health post level. We also searched web-based sources. The following documents were included in the review process: CHA national strategy, newsletters, job descriptions, reports and CHA implementation guides as well as three recent studies on CHA programme in Zambia.

### Data analysis

Interviews were recorded digitally and later transcribed verbatim by the first author and reviewed by all authors. Data analysis followed thematic analysis which ‘is a method for identifying, analysing and reporting patterns (themes) within data. It minimally organizes and describes data set in (rich) detail and goes further to interpret various aspects of the research topic’ [31].

The first step in analysing data was the development of codes. The first author developed initial codes after reading the transcripts several times to develop a sense of the whole dataset. The coding process was carried out with the use of NVIVO version 7 (QSR Australia). The codes were shared with the other authors for review. Codes were then grouped into categories - groups of content that share a commonality - and these were then developed into broader themes. This involved interpreting the categories for their underlying meaning, and grouping categories according to patterns as reflected in Table 1.

**Table 1:** Selected codes, categories and themes

**Codes**	**Categories**	**Themes**
-Advertising CHA position	CHA recruitment process	Becoming a Community Health Assistant
-CHA selection committee		
-Attending CHA interviews		
-Training in community health services	Motivation for becoming A CHA	
-Working as community health worker		
-Working as traditional birth attendant		
-Member of neighbourhood health committee		
-Desire to solve community health problems		
-Bringing health services close		
-Being part of community		
-Wanting to get a better job		
- Desire to improve skills		
-Hope to enhance experience		
-Conducting tasks at health post	Enhancing professional skills	CHA Experiences at the health posts
-Participating in meetings at health post		
-Restricted duties at health post	Exclusion of CHAs from the health post	
-Misallocation of tasks		
-Limited sharing of resources		
-Supervisor’s knowledge of programme	Supervision and work performance	
-Participating in supervisory meetings		
-Review of CHA reports		
-Payment processes	Monthly incentives and work motivation	
-Type of services provided in the community	Addressing health problems in the community	Experiences in the Community
-Support from the community		
-Respect from the community		

Finally the themes were cross-checked with the interview transcripts in order to ensure that they were applied to relevant responses found within and across the interviews. The focus was placed on identifying, summarising and retaining the patterns and similarities, differences and new emerging themes. Data from in-depth interviews were then triangulated with other sources such as the information gathered through observations and review of documents. The triangulation involved assessing the consistency and potential variations of findings by comparing data patterns across the material generated by different methods. This process showed that the major issues raised by CHAs, such as participation in duties at the health post (including meetings), availability of supplies, as well as trained supervisors at the health post, were consistent across the different types of data.

### Ethics

Ethical clearance to conduct the study was obtained from the University of Zambia Biomedical Research Ethics Committee (IRB 0001131 of IORG 0000774, reference number 009-10-11). Permission was also obtained from the MoH to conduct the study. During the data collection process, verbal consent was obtained from the CHAs before interviewing them. All the study objectives were clearly explained to them and CHAs were informed that they were free to withdraw from the study at any point. Informants were also assured that none of their personal details or other identifiers would be included during the analysis and subsequent publication of the findings. By withholding respondents’ personal details, it is not possible for readers to attribute views or statements to specific CHAs.

## Results

This section describes major issues which emerged from the interviews with CHAs regarding their experiences working at the health post and in the community. The findings section has been categorised into three broad areas: reasons for becoming a CHA; CHA experiences at the health post; and CHA experiences in the community. The section starts by outlining the characteristics of CHAs.

### Sociodemographic characteristics

Five CHAs were women and seven were men. Their ages ranged from 25 to 39 years. All CHAs had completed senior secondary school and were married. They all spoke Bemba (local language) and English. Further all CHAs were Christians and had resided in their area of operation (health posts) for not less than 5 years. About half of the CHAs had worked as community-based health workers prior to the programme.

### Becoming a community health assistant

#### Recruitment process

Discussions with CHAs showed that they were recruited following their response to the advertisement which was placed at the health posts, requesting eligible individuals to apply for the position of the CHA. The advert was put there by the MoH through the District Health Management Team (DHMT). A committee consisting of a representative from the DHMT, in-charge at the health post and the Neighbourhood Health Committees (NHCs) was established to shortlist and interview applicants. The CHAs reported that the recruitment process was quite competitive as on average about seven people attended interviews at each health post.

‘*I just saw the advert that they needed CHAs and that the qualification was a grade 12 certificate, [so] I decided to apply. We did the interviews in September 2010 and we were picked in May 2011. They were picking two people from each post.*’ (CHA 2, female).

We further asked CHAs what motivated them to apply for the position of CHA. As explained below, the reasons for applying for position centred on the position being related to their previous experience, career progression and a desire to contribute towards solving community problems.

### Motivation for becoming a CHA

#### Previous experience and knowledge

Most of the CHAs said that they were motivated to apply for the job because they were familiar with some of the tasks of the CHA position. Some CHAs had previously worked as community-based health workers while others had been through some short training on how to handle community health issues. Related previous training included psychosocial counselling, community mobilisation, testing for malaria and peer education on HIV/AIDS. Previous experience inspired CHAs to apply for the position as it not only helped them to understand the scope of the CHA position but also made them confident that they would get the position once invited for interviews.

‘*I worked as a community health worker for not less than 5 years before I saw the CHA advert. Thus I did not hesitate to apply for it because some of the responsibilities of the CHA seemed familiar.*’ (CHA 12, male).

#### Passion to serve the community and social bonds

Being residents of the communities, all CHAs stated that they were fully aware of the challenges that their friends, relatives and other community members experience, and that they viewed the CHA position as one way through which they would help resolve these problems. They indicated that some of the deaths that had occurred in the community (for example, from malaria and diarrhoea) could have been prevented if the community had sufficient trained health workers to provide the necessary services. In addition, having worked as community health workers before, some of the CHAs had developed strong bonds with health committees in the community and viewed this as an opportunity to further advance the collaboration.

#### Seeing beyond the CHA position

Discussions with CHAs suggested that most of them did not see their CHA position as an end in itself but rather a stepping stone for greater things. They reported that they had always dreamt of either becoming a nurse, environmental health technician, clinical officer or medical doctor. The desire to aspire towards something bigger than the CHA position was one of the key factors that helped some CHAs put in their best during the interviews and throughout the training. Analysis of the CHA strategy showed that it supports career progress, although it does not clearly state exemptions or support that would be accorded to those with aspirations of becoming a nurse or clinical officer.

‘*I have always wanted to be a nurse in future. So this is a good opportunity for me to gain experience in dealing with health matters. I am happy because the community is already calling me nurse.*’ (CHA 10, female).

### CHA experiences at the health posts

#### Perfecting and broadening professional practice

Having completed school, CHAs were immediately deployed at the health posts and assigned tasks. Review of the CHA job description and interviews showed that CHAs should perform a wide range of activities at the health post which include screening patients (that is, taking vital signs). They also test for malaria, and diagnose and treat minor illnesses such as diarrhoea and respiratory tract infections.

‘*Here where we are sitting in is the screening room, so I sit here and screen patients and sometimes I dispense drugs. Sometimes I test for malaria.*’ (CHA 9, male).

Discussions with CHAs showed that because of the limited number of trained staff at health posts, it was resolved that CHAs should spend more time at the health posts than in the community. CHAs at a few health posts were happy with this arrangement for several reasons. First, it provides them the opportunity to perfect their skills under the close supervision of the nurses, as not all CHAs had an opportunity to adequately practice at the training school. Others found this as an opportunity to rest as community work often involved travelling long distances using a bicycle.

‘*The thing is when we started work, we were supposed to be 20*% *here and 80*% *in the community but this changed because the people here are not trained to handle drugs. It is only the in charge who is trained. So we were told that we should at least help and work 50*% *at the health post.*’ (CHA 6, male)*.*

In addition, CHAs in a few health posts reported that working at the health post provided them with the opportunity to learn new things. For example, some CHAs had learnt how to use other relevant drugs to treat illnesses when the medicines that they are familiar with - the ones they learnt at the school are out of stock. They reported that this additional knowledge was very useful, as getting new supplies is not easy due to long distances between the health post and the main drug storage facility in town.

‘*He (supervisor) has taught us on how to use other medicines when the medicines that we learnt at the school are not available, for example treatment for eyes which often runs out quickly.*’ (CHA 11, male).

### Exclusion of CHAs from the health post

While CHAs in some of the health posts had very good work experience at the health posts, others did not. Those who complained of facing difficulties cited limited involvement in various tasks at the health post. A lack of full involvement in the meetings and omission from the staff lists were other frustrating factors. Furthermore, failure by a few health posts to allow CHAs to dispense drugs or to be in the dispensary on their own made some CHAs feel as if they were not fully trusted. Observations confirmed that CHAs were not always on the list of staff at the health posts or invited for meetings. A CHA narrated to us some of the challenges that he encountered in performing his duties at the health post:

‘*It’s like they are underrating us. You can tell by looking. You can see on the board - there is a list of members of staff here - and we are not included on this list. We are considered as non-staff. Even if you come early and sit at the dispensary, the cleaner will come and stand behind you and wait for you to move out so that he can dispense medicines, and if you don’t move he will tell you to go… Sometimes… you will find there are a lot of patients here and when they go for lunch, you may want to help clear the queue - but they will lock up the dispensary and leave you out in the screening room - because they don’t trust us with the medicines.*’ (CHA 3, male).

Interviews with CHAs further showed that this exclusion or competition also took the form of misallocation of tasks. It was reported that some support staff delegated their responsibilities to CHAs while they performed clinical duties. Discussing with a CHA at the health post, she narrated to us how one morning she reported for work only to be requested by a cashier to sweep the health post while the cleaner himself proceeded to screen patients.

Furthermore, some of issues which brought dissatisfaction among CHAs and competition between CHAs and existing staff had to do with discrimination with regards to the sharing of financial resources. It was reported that participating in some community immunisation programmes attracts extra allowances. However, only a few staff can participate in these programmes due to limited finances. It was therefore reported that some staff were concerned that CHAs would compete with them for these limited opportunities. Furthermore, health posts receive about 1,200 kwacha (about 175 US dollars) petty cash every month, an amount which was not sufficient to accommodate the needs of new staff. Below is in an illustration of how difficulties in sharing limited financial resources resulted in the old group of staff (support staff) classifying the new group of workers (CHAs) as belonging to the ‘other’ (being under Clinton Health Access Initiative) and not part of ‘them’ (staff under the MoH).

‘*Like this centre gets 1,200 kwacha for general maintenance at the health post every month. We just asked them to give us part of the money since we have delays in our salaries. We needed the money to repair our bikes. But they said no, this is programme is not under the Ministry of Health, you are sponsored by Clinton Health Access Initiative (CHAI), so go and ask CHAI for the money.*’ (CHA 6, male).

Limited pharmaceutical and financial resources, inadequate understanding of the role and nature of the CHA programme and communication challenges between various stakeholders contributed to competition between CHAs and existing staff. Further, fear by some supervisors of being moved to other health centres following the deployment of CHAs at the health posts contributed to this competition.

‘*The only thing that was bad was the misconception supervisors had when we came. They thought that they would be shifted to other areas. So they felt threatened and tried to frustrate us at first. For example, our supervisor would say that you are not supposed to handle drugs when you are at the clinic, just observe, then go and work in the community. But after a while she saw we were complementing each other.*’ (CHA 6, male).

### The role of effective supervision in promoting work performance

Further analysis showed that clear definitions and an understanding of staff responsibilities at the health posts are essential in promoting good work performance. It was reported that the ability by the supervisor to effectively translate the knowledge acquired from the supervisory course into proper definition of tasks at the health posts was essential in enhancing CHA work performance. For instance, in a few health posts, supervisors had managed to involve their CHAs in tasks at the health posts by including them in the meetings and regularly holding discussions with each CHA aimed at reviewing CHA reports, targets, challenges as well as success stories.

‘*We relate well with everyone here. Because when we came, our supervisor called a meeting and explained our responsibilities. So, when we report for work, we all take our places.*’ (CHA 11, male).

Although supervisors in some health posts had taken the initiative to include CHAs in the meetings and regularly discuss their daily experiences, others did not. The differences in the level of engagement between the supervisors and CHAs were attributed to misconceptions about the role of CHAs at the health post, and limited knowledge by some supervisors of the CHA programme. Limited knowledge was the result of transfers of supervisors who had been trained in the programme to other health posts. One illustration of limited knowledge of the programme was about the process of setting goals.

‘*We need to set goals on how we work each day, and when I told the in-charge, she said she didn’t know how to set the goals, so I just work according to the way I can manage as long as am just reporting.*’ (CHA 5, female).

Delayed communication of important information to CHAs by a few supervisors also demoralised CHAs. For example, it was reported that some supervisors shelve important documents instead of giving them to CHAs upon receiving them from the MoH.

‘*So it’s today that I have found a certain CHA implementation book. I asked her (supervisor) when it came and she said a long time ago. There are also other materials that came in October last year and we are only seeing them now in May.*’ (CHA 6, male).

### Monthly incentives and work motivation

Although CHAs had signed contracts with the MoH which stipulated that they would be paid monthly allowances, and they were generally satisfied with the monthly incentive rate which they had signed up for, seven had been paid for 5 months of the first 9 months they had worked at the time of interview, while the rest had not received anything at all. Discussions with CHAs showed that they were not sure about the causes for the delay or non-payment of the incentives. Furthermore, they were not sure where to turn to for answers to this problem. It was reported that many other CHAs in the country had also been affected by this problem, and that at in May 2013, when CHAs were invited for a 2-week refresher course at the training school in Ndola district, they held a peaceful demonstration to express their displeasure over the matter.

‘*We held a demonstration at the college demanding payment of salaries because many of us have not been paid. It is like only a few lucky ones have been paid. It is not clear why it is like this.*’ (CHA 8, male).

The situation was very frustrating for CHAs as they could not manage to meet the basic needs for their families. This is because they spend 5 days in a week doing CHA duties and have no time to venture into other income generating alternatives. This financial problem had made it difficult for CHAs to renew their practising certificates. What worsened the situation was that it had become difficult for them to borrow money from the community, as they believed it was not good for professional health workers to continuously ask for money from the community. They felt that doing so would potentially undermine the high status which had been accorded to them by the community through giving them the title of ‘nurse’. Their frustration was compounded by the demand by some patients/clients for more input from them, due to the view that government was paying them monthly salaries when in reality they were not getting paid.

‘*When there are long queues, people would shout at you for being slow and say you are delaying us after all you are paid a lot of money to serve us. But it’s depressing because we have not been paid for 9 months. What is even worse, it is difficult for us to ask for money from the community.*’ (CHA 4, female).

### Experiences in the community

#### Translating the dream to serve the community into reality

The first activity for CHAs in the community was a mapping exercise. This exercise was aimed at identifying key health problems that people face. While doing mapping, CHAs also developed a register of community members. Review of the fieldwork reports showed that CHAs issue receipts to households every time they visit the household as confirmation of the visit. Other activities are promoting the construction of toilets and digging of refuse pits.

‘*We did a community diagnosis and mapping exercise. We had to go around the catchment area and spot out the health problems. We found a lot of cases of malaria, diarrhoea and people not having pit latrines. Then we started putting interventions starting with the furthest catchment area that is across the pontoon about 8 to 12 kilometres from here. For example we gave information on how to purify drinking water because most of the people here draw water from the stream.*’ (CHA 1, male).

Seeing things progress well was something that motivated CHAs to continue working despite facing some challenges. One of the CHAs who thinks that things have really gone well in his site cited an increase in the number of mothers delivering babies at the health post, people using family planning methods in the community, and adhering to practices of maintaining good sanitation standards as some of the factors that encouraged her.

‘*Because of our activities, the number of people using family planning methods has increased from 20*% *when I started to 50*%*, [and] the number of women delivering babies at the health posts has increased from about 5 or 6 per month to about 15 to 20 per month.*’ (CHA 12, male).

Sitting and freely taking to the people as well as being in control of one’s schedule were also very exciting for most CHAs. Analysis of data also showed that the ample space CHAs had to exercise their skills and demonstrate their expertise to the community was very gratifying to them.

‘*It’s very good out there (community), you sit with them and talk to them and they start asking you questions.*’ (CHA 5, female).

### Challenges in the community

Although many of the CHAs told positive stories about their experiences in the community, a few CHAs complained that some NHC members had limited confidence in their capacity to perform their duties. CHAs attributed this to their not being fully involved in the activities at the health posts. It was reported that some community members had noticed that some CHAs were not very active at the health posts. However, in a few cases, support staff’s inability to clearly articulate the role of CHAs contributed to this limited confidence in CHA services.

‘*The way we are working with the neighbourhood health committee, we are not comfortable… they are not regarding us as trained staff. So they regard the cashier, watchman and cleaner as people who have better information. Sometimes even when they ask the staff here they say they don’t even know us and the community will sometimes reject us as a result. There’s nothing we can do because when they come here (health post), they find us not working.*’ (CHA 3, male).

Related to this, few CHAs reported limited support from their NHC members and community-based health workers in mobilising communities and conducting health promotion activities. This was attributed to the poor introduction of the CHAs to these local structures and committees. Further, the complaint by some community-based health workers who felt unfairly treated because they are not entitled to the monthly incentives also affected community support. It was reported that some community-based health workers were not willing to help the CHAs conduct their activities because of this non-entitlement to incentives.

‘*Even when you call for the meeting and ask the neighbourhood health committee to mobilise people for sensitisation programmes, some do not agree. Some have also withdrawn their services. They tell us that they cannot help us since we are paid and they get nothing.*’ (CHA 9, male).

Inability by most CHAs to carry medicines to the community due to limited drug supplies at the health posts also limited their performance. It was reported that some people in the community, especially the young and old, were greatly affected by this limited availability of drugs at community level. For example, a CHA reported that she was not able to treat a malaria case after diagnosing it due to limited drugs.

Despite these challenges, CHAs stated that they would still continue working. Most of them reported that stopping work would betray the good ties that they had with the community. Others cited the support from supervisors and the self-awareness of the need and value of improving their professional skills and experience as some of the factors that kept them going. Overall, they were hopeful that a lasting solution would be found to their problems.

‘*We work because we have a heart for the people but we need motivation. At the training school, we were told that people (from the Ministry of Health headquarters) would come to find out how we are working. I think that after that, things will be better.*’ (CHA 1, male).

## Discussion

This study has explored the motivation for becoming CHAs, their experiences of working in a rural district in Zambia, and how these experiences affected motivation to work. The findings reflect the situation at the early stage of the programme pilot, and this paper therefore aims to contribute to the process of identifying the challenges in this early phase as well as providing viable recommendations for addressing these challenges.

Our results show that the process of becoming a CHA was influenced by a combination of individual factors which include their assessment of the level of qualifications required for the job, community connectedness and a passion for service. Factors that influenced CHAs’ work motivation included power relations between CHAs and other staff at the health post, health post work culture and the structure as well as the level of support received from the community. Below we discuss the findings from the perspective of the three key factors identified by the WHO that influence performance of health workers: characteristics of the health workers; the health system; and the population served by the health workers [4].

### Individual characteristics and the choice of becoming a CHA

Individual characteristics that motivated people to become CHAs included the desire to improve knowledge and experience in health service delivery, the possibility of getting a better job in future, as well as an aspiration to help resolve community health problems. This finding is consistent with other studies on community-based health worker programmes in low and middle income countries [15,32,33], as well as a recent study on the CHA programme conducted in 48 districts in Zambia [23].To reinforce the feeling of community connectedness, community members have been included in the committee that recruits CHAs. Furthermore, only people who reside within the community are selected. This is intended to help ensure that those selected relate well with and understand the community, which is the cornerstone of the community-based health worker model [8,34]. In addition, locality-based ‘selection of candidates’ [35] is generally believed to contribute towards promoting retention of staff in rural areas, and it also minimises geographical barriers to service provision [12,36]. Overall, these processes could improve performance of CHAs by triggering the ‘anticipation of being valued by the community, a perception of improvement in social status, and having a valuable social role and accountability to the beneficiaries’ [37].

### Health system characteristics and motivation to work

Health systems characteristics such as work culture, supervision and availability of resources have also influenced CHAs’ motivation to work. Health posts with an inclusive work culture appeared to have positively influenced CHA work motivation. Our results are consistent with the findings from a recent process evaluation of the pilot phase of the CHA programme in four districts in Zambia which showed that health systems characteristics have affected CHAs’ ability to work [38]. The study showed that staff in the districts regarded CHAs as a special group of workers who should not use the drugs held at the health posts. Some supervisors thought that CHAs would receive CHA-specific drug kits from the MoH national headquarters. As a result, supervisors refused CHAs access to drugs, thereby ‘forcing CHAs to mainly focus on providing health education at community level rather than on providing curative services’ [38]. The study further showed that limited involvement of the CHAs was partly due to inadequate communication or interaction between CHAs and existing staff at the health posts which was attributed to not all CHAs being formally introduced to the health post staff by representatives from the District [38]. This in turn has contributed to insufficient understanding of the role of CHAs and potentially contributed to limited perceptions of CHAs as being fully part of the health system. Our study, however, goes further to demonstrate that limited resources triggered competition among staff in some health posts a situation which did not only result into exclusion from duties but also misallocation of tasks in certain instances. Studies conducted on similar community-based health worker programmes (for example, in Ethiopia, India, Pakistan and Brazil) have also indicated that work culture, supervision and availability of resources play a crucial role in influencing community-based health workers’ involvement in work [6,11,10,14].

Full involvement in duties is important because it provides possibilities for improving skills, which is essential in enhancing personal feelings of empowerment and satisfaction [39]. This sense of self-empowerment facilitates good work performance as it strengthens a person’s belief (confidence) about their capabilities (self-efficacy) to execute tasks [37]. Regular supervision and involvement in meetings is likely to influence performance because these activities provide opportunities for interactions, clarifications and receiving feedback, which can act as a social glue for holding staff together [40]. Conversely, limited involvement and supervision negatively affects CHAs’ motivation to work because supervisors can only confidently delegate tasks to people about whose skills they are sure [25,41,42]. Similarly, employees are likely to be committed if they perceive their supervisors as valuing their skills, as this gives them confidence to effectively undertake tasks [1,43,44]. Our findings therefore provide further evidence of the far-reaching influence of supervision on work performance, and specifically in institutionalised community-based health worker programmes.

Limited knowledge of the programme, prioritisation of CHA tasks and some supervisors’ busy schedules contributed in some cases to inadequate supervision in Kapiri Mposhi. These findings are similar to results from a recent process evaluation of the CHA pilot phase in four other districts [38]. However, in addition to these, our study further shows that social and economic (livelihood) insecurities of staff, which could have been triggered by a fear of losing their positions to CHAs, seemed to have contributed to the exclusion of some CHAs from the health posts to which they had been assigned. The findings suggest the existence of a social structure which constrains CHAs from being innovative, or to perfect and gain additional skills. Such a limiting social structure could affect motivation and performance because it limits an individual’s agency which is ‘the capacity to transpose and extend schemas to new contexts’ [45]. Further, an excluding structure has negative implications on CHAs’ ability to confidently define their professional identity. This is because significant others, in this case existing staff at the health post, play a major role in building CHA identity through feedback processes. It is important to note that ‘feedback about one’s position can provide a sense of security or sense of threat to self’ [46]. Negative feedback in the form of doubting a CHA’s expertise, not trusting CHAs with drugs, or misallocation of tasks may, over time, make CHAs perceive themselves to be less competent to perform their tasks that they really are. The importance of closely analysing the effects that words, actions and expressions can have on people has previously been noted, as these may result in the classification of individuals as ‘“us” and “others”’ [47]. This classification process could have led to the reported refusal by some staff to share resources with CHAs in some health posts, as they perceived them as belonging more to the CHAI than to the MoH. This limited sense of belonging to their health post could have serious implications on CHAs’ contributions at the health post, as well as undermining their ability to deliver quality health services at the local level.

Addressing the problem of supervision may require grooming CHAs to supervise others, an approach that has been used in a community-based health worker programme in Bangladesh [42]. This may therefore require creating formal supervisory positions into which CHAs with skills and experience in specific domains can progress. This process could develop supervisors who effectively relate to the expectations, pressures and context in which CHAs perform their duties. Further, this could help create a career pathway which is essential for facilitating both motivation to work and staff retention [33], in a programme which intends to train about 5,000 CHAs.

The Health System’s failure to regularly pay allowances also dissatisfied CHAs. Review of documents showed some of the causes of this delay include setbacks in administrative processes and limited communication from the MoH to the district level which resulted in‘leaving districts unsure of what to communicate to supervisors and CHAs’ [38]. However, despite their financial problems, interviews from Kapiri Mposhi as well as data from other districts in Zambia [23,38] showed that CHAs continued reporting for work. This probably provides additional evidence on the role of personal attributes such as dedication, reliability, persistence and community connectedness [33,39], in facilitating work motivation among community-based health workers integrated in the health system. Several studies on community-based health workers have demonstrated that non-financial issues such as personal attributes, setting clear responsibilities, improving grievance procedures, appropriate job aides, resources/supplies and mentorship systems at the local level could improve work motivation [6,10,11,14]. Non-financial factors may improve work motivation as they may trigger ‘a sense of relatedness with the local public health services, and thus accountability towards the system, a sense of credibility and legitimacy of being part of the local public health services, an anticipation of being valued by the local public health services as well as an assurance that there is a system for back-up support’ [37]. These processes could also help normalising work relationships [48], and possibly strengthen trust among workers [49].

### The role of the community in shaping CHA motivation to work

Appreciation and respect by the community on account of their being provided with health services also motivated CHAs to work, a finding consistent with a recent study conducted in other districts in Zambia [23]. Having a network of constructive social support improves work performance at the local level because it strengthens relationships with the community, which in turn improves feelings of recognition as well as possibilities for participation and autonomy [50]. This resonates with findings on Brazilian community health agents where the subject of respect and support from the community often dominated the responses when asked why they liked their jobs [51].

On the other hand, like a recent study [38], our study indicated that limited work time in the community as well as a lack of clarity of roles and scope of work of CHA dissatisfied the community and have the potential of undermining the community’s confidence in CHA activities. In addition, this study also indicates that failure to treat common illnesses due to CHAs’ inability to carry drugs to the community attracted negative feedback from some community members, which may in turn undermine the community’s trust and confidence in the health services more broadly. Such negative community perspectives could reduce utilisation of services if not addressed [42,52]. Further, the concept of paying CHAs’ incentives was not well received by some existing community-based health workers, who were reported to have withdrawn their services due to the fact that they are expected to work on a purely volunteer basis. Limited support by these community-based health workers also appeared to have affected CHA performance, as the catchment areas are too vast for them to effectively handle on their own. However, addressing these challenges many not necessarily require putting all categories of community-based health workers on the government payroll. Considering the difficulties in paying CHA incentives and the role of non-financial incentives in promoting work motivations, we agree with Schneider [33] that relatively loose processes surrounding the selection and deployment of community-based health workers and voluntary forms of participation may potentially be advantageous. Improving non-monetary incentives such as providing them with materials that identify them as community-based health workers (badges, t-shirts, and so on), frequent refresher training, supportive supervision [53] and closer links to CHAs, could motivate this group of health workers to collaborate more effectively with CHAs.

In summary, by simultaneously putting in place multiple, locally tailored incentives, such as those identified above, at the individual, community and health system levels [37], CHA motivation to work could be effectively improved.

### Limitations of the study

One of the limitations of the study was that we were not able to conduct the observation of CHA activities in the community. This denied the study some important perspectives on motivation of CHAs to conduct their duties in the field. Furthermore, the study setting was only one district, a situation which limits generalisability of the findings to other districts. In addition, the perceptions of these CHAs are only one part of the picture, and their views need to be complemented with views of their supervisors, health staff in the facilities and community members. It is also possible that the half-day observations conducted at the health posts may have engendered a Hawthorne effect, whereby some aspects of people’s behaviour may have been modified through their being aware that they were being observed.

However, even though generalisability was not the intention, the rich description of phenomena, our multiple methods of data collection and our triangulation of data all helped in developing an account that we believe provides a valuable contribution to the knowledge base on factors that shape work motivation among institutionalised community-based health workforce.

## Conclusions

We sought to explore the motivation to becoming CHAs, their experiences of working in a rural health district in Zambia, and how these experiences shaped their motivation to work. Personal characteristics such as previous work experience and knowledge as well as a passion to serve the community motivated people to become CHAs. Health system characteristics such as an inclusive work culture in some health posts positively affected CHA motivation to work. However, exclusion of CHAs from other health posts, misconceptions of CHA roles, limited supervision and poor prioritisation of CHAs tasks by some supervisors, as well as non- and delayed payment of CHA incentives all reduced motivation and constrained CHAs’ ability to do a good job. Community attributes such as support and respect for CHA services triggered a sense of recognition and connectedness to the community in CHAs, which in turn positively impacted their work motivation. On the other hand, limited drug supply and a lack of support from some existing community-based health workers negatively affected CHAs’ ability to work at the community level. Simultaneously and adequately considering the multiple individual, health system and community factors that promote work performance among community-based health workers in institutionalised programmes at the local level could improve the contribution of CHAs towards improving health outcomes. By systematically highlighting context-specific realities of CHA involvement in duties at the health post and in the community, the study could provide useful insights to the MoH for the CHA programme scale-up phase. Furthermore, our findings are also relevant to other low and middle income countries that intend to integrate community-based health workers into the health system.

## Abbreviations

ASHA: Accredited social health activist; CHA: Community health assistant; HEW: Health extension worker; HRH: Human resources for health; LHW: Lady health worker; MoH: Ministry of Health; NHC: Neighbourhood health committee.

## Competing interests

The authors declare that they have no competing interests.

## Authors’ contributions

All four authors contributed towards the study design. JMZ, JK and AKH carried out the data collection. All authors analysed the data. JMZ drafted the manuscript and all authors contributed towards revision of the manuscript. All authors read and approved the final manuscript.
